# Postural Changes and their Influence on Functional Behavior of the Great Arteries

**DOI:** 10.36660/abc.20190691

**Published:** 2019-12

**Authors:** Luiz Aparecido Bortolotto

**Affiliations:** Unidade Clínica de Hipertensão do Instituto do Coração do Hospital das Clínicas da Faculdade de Medicina da Universidade de São Paulo, São Paulo, SP - Brazil

**Keywords:** Aorta/physiopathology, Hypertension, Hypertrophy,Left Ventricular, Blood Flow, Pulse Wave Analysis, Stending Position, Gravitation, Aging.

The large arteries, especially the aorta, are known to play a major role in the circulation of blood flow both during ventricular ejection and during diastole, thus providing the blood supply required by the organs. Additionally, the damping function exercised by the aorta plays a fundamental role in central hemodynamics, providing adequate ventricular-arterial coupling during the cardiac cycle.^1^The loss of this function due to increased arterial stiffness plays a role in the development of hypertension and left ventricular hypertrophy and has been associated with the development of atherosclerosis and myocardial ischemia.^2^ Noninvasive methods for assessing the function of large arteries, such as pulse wave velocity (PWV) measurement, have provided a better understanding of the correlations of arterial stiffness with cardiovascular disease, and have been used as prognostic markers in different populations.^3^However, due to methodological limitations, all studies have been performed in the supine position, and the impact of postural changes, especially orthostasis, has not been evaluated on these vascular properties. Thus, the study by Elias Neto et al.^4^ published in this issue, as the first to evaluate the effect of orthostasis on the aortic functional properties in normotensive and hypertensive individuals not treated by the aortic PWV measurement in humans, provides important information to understand the role of these properties in the physiological adaptation of central hemodynamics in relation to the gravity effects. The authors evaluated nearly 100 individuals with no evident cardiovascular disease by measuring carotid-femoral PWV performed in the supine and standing positions after the 70° tilt test and demonstrated a significant and sustained increase in PWV throughout the tilt test, in both young and older individuals. Interestingly, although there was a direct and significant association between baseline and post-orthostasis PWV values and systolic blood pressure (SBP ), SBP remained unaltered or showed a slight decrease during the tilt test and, therefore, the increase in PWV during inclination occurred by other mechanisms of circulatory adaptation, including an increase in peripheral arterial resistance (indirectly suggested by the observed increase in mean BP ) and changes in circulatory dynamics promoted by the gravitational force, which are related to the differential structural and geometric characteristics of the aorta throughout its trajectory. In a study with a smaller number of individuals and another methodology for PWV assessment, the authors also found an increase in PWV with the tilt test and attributed this increase to increased hydrostatic pressure and greater sympathetic activity.^5^

Due to gravitational effect, blood flow increases in the more distal arterial segments, which have smaller diameter and less elasticity, causing an increase in PWV and also in BP in the infradiaphragmatic territories.^6^ The consequence of this increase in carotid-femoral PWV would be the early return of the retrograde wave to the heart, increasing pulse pressure at the aortic root, which could contribute to maintaining a more adequate cerebral blood flow with the biped position assumed by humans. It is noteworthy that this same mechanism of early wave reflection in the aortic root is one of the main factors responsible for SBP increase in the elderly, as a consequence of the increase in arterial stiffness observed with aging.^7^ Evaluating the results of the study by Elias Neto et al., the PWV of young individuals after orthostasis showed values similar to those found in the elderly in the dorsal position, corroborating the role of increased PWV with increased retrograde wave in the individual’s adaptation to the orthostatic position.

However, it is still necessary to recognize the role of this adaptive circulatory phenomenon to the orthostatic position in the development of cardiovascular pathologies, such as arterial hypertension and atherosclerosis. One could speculate, for instance, whether an exacerbated response of this adaptive mechanism, with an exaggerated increase in PWV due to orthostasis, could participate in the mechanism of arterial hypertension in some situations, such as isolated spurious systolic hypertension of young individuals, partially explained by the increase in the pulse wave amplification phenomenon between the aorta and the brachial artery.^8^

Recently, some studies have evaluated the influence of gravity on arterial stiffness and the role of these changes in orthostatic adaptation to the absence of gravity.^9,10^ In one of these studies, the authors demonstrated that astronauts who showed more tolerance to orthostatism after a prolonged period in space showed an increase in arterial stiffness manifested by increased pulse velocity, while intolerant individuals showed increased arterial distensibility.^9^ These data reinforce the participation of increased PWV in the process of adaptation to gravity.

As mentioned by the authors, the study has some limitations, making some hypotheses just speculative ones, such as the participation of greater sympathetic activity in this process, since it has not been directly evaluated. Another limitation not mentioned by the authors, but which may show differences in result interpretation, is the predominance of males in the studied population. It is well known that women have higher measures of arterial stiffness, especially earlier reflection waves, probably associated with smaller aortic diameter.^11^ These changes could interfere with the response of PWV to orthostasis in different genders, as seen in a small study with astronauts who remained 6 months without gravity, with women showing greater degrees of carotid stiffness than men after returning to the effect of gravity.^10^

Nevertheless, these findings provide important parameters for discussing the participation of large-vessel buffering properties in physiological adaptations to postural changes, which may provide new therapeutic strategies for clinical conditions where these adaptations are inadequate.
